# Maternal folate, one‐carbon metabolism and pregnancy outcomes

**DOI:** 10.1111/mcn.13064

**Published:** 2020-07-28

**Authors:** Tanja Jankovic‐Karasoulos, Denise L. Furness, Shalem Y. Leemaqz, Gustaaf A. Dekker, Luke E. Grzeskowiak, Jessica A. Grieger, Prabha H. Andraweera, Dylan McCullough, Dale McAninch, Lesley M. McCowan, Tina Bianco‐Miotto, Claire T. Roberts

**Affiliations:** ^1^ Robinson Research Institute University of Adelaide Adelaide South Australia Australia; ^2^ Adelaide Medical School University of Adelaide Adelaide South Australia Australia; ^3^ Department of Obstetrics and Gynaecology Lyell McEwin Hospital Elizabeth Vale South Australia Australia; ^4^ Department of Obstetrics and Gynaecology University of Auckland Auckland New Zealand; ^5^ School of Agriculture Food and Wine, Waite Research Institute University of Adelaide Adelaide South Australia Australia; ^6^ College of Medical and Public Health Flinders University Bedford Park South Australia Australia

**Keywords:** folate, folic acid, maternal diet, neonatal outcomes, one‐carbon metabolism, pregnancy outcomes, SNP

## Abstract

Single nucleotide polymorphisms and pre‐ and peri‐conception folic acid (FA) supplementation and dietary data were used to identify one‐carbon metabolic factors associated with pregnancy outcomes in 3196 nulliparous women. In 325 participants, we also measured circulating folate, vitamin B12 and homocysteine. Pregnancy outcomes included preeclampsia (PE), gestational hypertension (GHT), small for gestational age (SGA), spontaneous preterm birth (sPTB) and gestational diabetes mellitus (GDM). Study findings show that maternal genotype *MTHFR A1298C*(CC) was associated with increased risk for PE, whereas *TCN2 C766G*(GG) had a reduced risk for sPTB. Paternal *MTHFR A1298C*(CC) and *MTHFD1 G1958A*(AA) genotypes were associated with reduced risk for sPTB, whereas *MTHFR C677T*(CT) genotype had an increased risk for GHT. FA supplementation was associated with higher serum folate and vitamin B12 concentrations, reduced uterine artery resistance index and increased birth weight. Women who supplemented with <800 μg daily FA at 15‐week gestation had a higher incidence of PE (10.3%) compared with women who did not supplement (6.1%) or who supplemented with ≥800 μg (5.4%) (*P* < .0001). Higher serum folate levels were found in women who later developed GDM compared with women with uncomplicated pregnancies (Mean ± SD: 37.6 ± 8 nmol L^−1^ vs. 31.9 ± 11.2, *P* = .007). Fast food consumption was associated with increased risk for developing GDM, whereas low consumption of green leafy vegetables and fruit were independent risk factors for SGA and GDM and sPTB and SGA, respectively. In conclusion, maternal and paternal genotypes, together with maternal circulating folate and homocysteine concentrations, and pre‐ and early‐pregnancy dietary factors, are independent risk factors for pregnancy complications.

**Key messages**

- Both parental genotypes in one‐carbon metabolic pathway genes influence pregnancy outcomes.
- Low consumption of green leafy vegetables and fruit increases risk for several pregnancy complications, whereas fast food consumption increases risk for gestational diabetes mellitus.
- Higher circulating folate levels increase risk for gestational hypertension and may be associated with gestational diabetes.

## 1 INTRODUCTION

Adverse pregnancy outcomes, such as preeclampsia (PE), gestational hypertension (GHT), infants born small for gestational age (SGA), spontaneous preterm birth (sPTB) and gestational diabetes mellitus (GDM), can affect up to one in two pregnancies and have multifactorial aetiology and substantial maternal and neonatal morbidities (Roberts, 2010; SA Health Pregnancy Outcome Unit, 2017). Multiple factors can affect pregnancy health, including maternal nutrition, age, lifestyle and socio‐economic status, as well as maternal and paternal genetics. Importantly, these factors often act in concert with one another.

**FIGURE 1:**
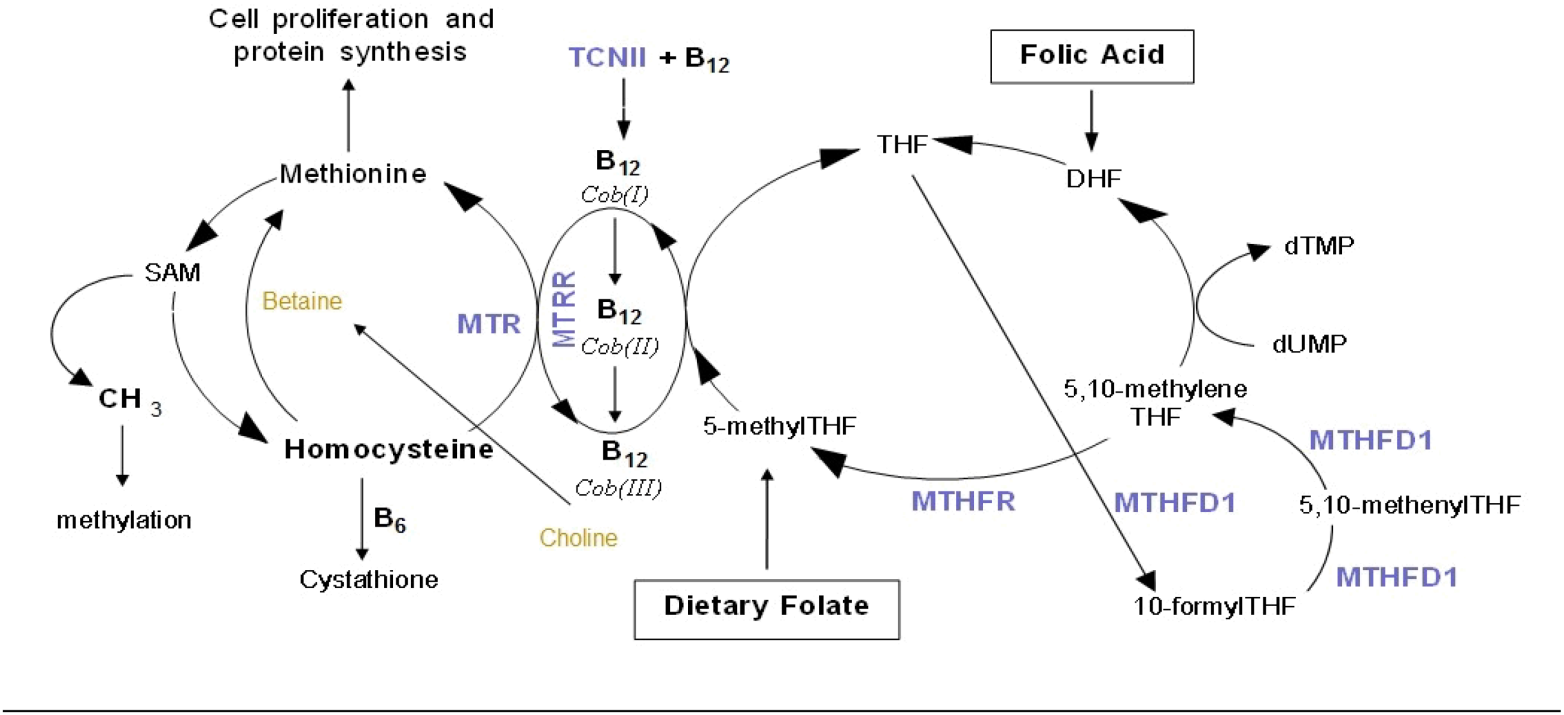
The one‐carbon metabolic pathway. Folate, as 5‐methyl‐THF, serves as the substrate for metabolic transformation of homocysteine to methionine via MTR. Methionine is converted to *S*‐adenosylmethionine (SAM), which serves as the principal methyl donor for methylation. Vitamin B12 acts as an intermediate methyl carrier during the MTR‐catalysed re‐methylation of Hcy to methionine, cycling between two states, cobalamin (I) and methylcobalamin (III) via TCN II. Cobalamin (I) is a strong reductant that can be oxidised to produce inactive cobalamin (II), which undergoes reductive methylation to methylcobalamin (III) via the MTRR enzyme using SAM as the methyl donor. Choline derived betaine can also serve as a methyl donor for the conversion of Hcy to methionine. **B6**, vitamin B6; **B12**, vitamin B12 (cobalamin, in various oxidative forms); **CH**_**3**_, methyl group; **DHF**, Dihydrofolate; **Hcy**, homocysteine; **MTHFR**, methylenetetrahydrofolate reductase; **MTR**, methionine synthase; **MTRR**, methionine synthase reductase; **SAM**, S‐Adenosylmethionine; **SAH**, S‐adenosylhomocysteine; **TCN II**, Transcobalamin II; **THF**, tetrahydrofolate. Modified after Furness et al. (2008)

The maternal diet and nutritional stores provide nutrients for the developing embryo and fetus. These nutrients underpin growth by supporting high rates of DNA replication and cellular proliferation that take place during fetal life (Maloney & Rees, 2005) and preventing oxidative stress and inflammation, which have been associated with aberrant placentation and pregnancy complications (Bhupathiraju & Tucker, 2011; de Jonge et al., 2011; Field, van Aerde, Drager, Goruk, & Basu, 2006; Furness, Dekker, & Roberts, 2011; Potdar et al., 2009; Redman & Sargent, 2010; Sorokin et al., 2010). Folate (vitamin B9) is a dietary micronutrient, found in a variety of green leafy vegetables and fruits, which is required during pregnancy to support cell growth and healthy placental development and function. Folate serves as a donor of one‐carbon units for the remethylation of homocysteine (Hcy) to methionine and then to S‐adenosylmethionine (SAM), the universal methyl donor that affects systemic gene expression and cellular methylation potential (Bailey et al., 2015; Furness, Fenech, Khong, Romero, & Dekker, 2008; Hague, 2003) (Figure 1). Folate, together with vitamins B12 and B6 as cofactors, is required to maintain Hcy levels within a normal range (Brouwer et al., 1999; Holt et al., 2009; van Bussel et al., 2011), because at high concentrations, Hcy is pro‐inflammatory, leads to oxidative stress and has been associated with an increased risk for pregnancy complications (Dekker et al., 1995; Eskes, 2000; Forges et al., 2007; Poddar, Sivasubramanian, DiBello, Robinson, & Jacobsen, 2001; Singh, Thomas, Owens, Hague, & Fenech, 2015; Vollset et al., 2000).

When dietary folate consumption is inadequate, folic acid (FA) supplementation becomes an important determinant of circulating Hcy, with daily FA doses ranging between 200 μg and 5 mg being associated with 13–25% reduction in plasma Hcy levels (Homocysteine Lowering Trialists Collaboration, 2005). In fact, FA supplementation is recommended from 1 month prior to conception and during the first trimester of pregnancy to reduce the risk for neural tube defects; at least 400‐μg daily dose is recommended in Australia despite the 2009 mandatory fortification of FA in flour for bread production (The Royal Australian and New Zealand College of Obstetricians and Gynaecologists, 2014).

Folate and vitamin B status are not the only limiting factors for Hcy remethylation in the one‐carbon metabolic pathway. Polymorphisms in genes encoding enzymes involved in this pathway can also affect the conversion of Hcy to methionine, leading to increased circulating and cellular Hcy concentrations and DNA damage (Botto et al., 2003; Fenech & Ferguson, 2001) and possible adverse pregnancy outcomes (Furness et al., 2008; Laanpere et al., 2010; Nair, Khanna, Singh, & Singh, 2013; Tiwari et al., 2015). For example, methylenetetrahydrofolate reductase (MTHFR) converts 5,10‐methylenetetrahydrofolate to 5‐methyltetrahydrofolate, the biologically active form of folate that acts as a methyl donor for re‐methylation of Hcy to methionine. Single nucleotide substitutions in *MTHFR* are known to affect enzyme function thus limiting the bioavailability of folate and have been associated with pregnancy complications such as PE and intrauterine growth restriction (IUGR) (Chedraui et al., 2015; Lachmeijer et al., 2001; Liew & Gupta, 2015).

Our previous research has shown that genetic (Furness et al., 2008) and nutritional (Furness et al., 2013) one‐carbon metabolic factors at 20‐week gestation are associated with adverse pregnancy outcomes in a high‐risk pregnancy cohort. In the current study, we investigated the association between genetic, biochemical and nutritional one‐carbon metabolic factors at 15 ± 1 weeks of gestation with pregnancy complications in a low‐risk nulliparous cohort.

## 2 METHODS

### 2.1 Study design

The Screening for Pregnancy Endpoints (SCOPE) study was an international prospective cohort that recruited 5,628 nulliparous pregnant women from Adelaide (Australia), Auckland (New Zealand), Cork (Ireland), Leeds (United Kingdom), London (United Kingdom) and Manchester (United Kingdom). The aim of SCOPE was to develop screening tests to predict risk for pregnancy complications before women become symptomatic. A total of 3,196 women were recruited by the Adelaide–Auckland SCOPE centres between 2004 and 2008. We used data generated from the Adelaide–Auckland cohort (*n* = 3,196) to investigate associations between SNPs in the one‐carbon metabolic pathway, as well as preconception and early pregnancy maternal diets, with pregnancy outcome in women who developed a particular pregnancy complication compared with the remaining cohort. Study cohort included cases of PE (*n* = 222), GHT (*n* = 188), SGA (*n* = 298), sPTB (*n* = 156), GDM (*n* = 104) and other pregnancy outcomes (*n* = 354) that were not all mutually exclusive. There were 1,974 cases of uncomplicated pregnancies of which 209 were not tested for GDM and thus have an unknown outcome. Cases with any unknown outcome were excluded from data analyses. The results from this study are presented under study Part 1 in Section 3.

Serum biochemistry was analysed in a subset of 325 women from the Adelaide SCOPE cohort, for whom relevant blood biochemistry data were available. The aim was to determine whether circulating folate, vitamin B12 or Hcy, key players in the one‐carbon metabolic pathway, are associated with any of the variables or pregnancy outcomes tested in the 3,196 cohort. As this is a nested case–control study, which also includes cases of PE (*n* = 48), GHT (*n* = 46), SGA (*n* = 44), sPTB (*n* = 43) and GDM (*n* = 33) selected at random with 111 uncomplicated pregnancies selected as controls, the findings will be representative of the entire study population. All outcomes in the nested case–control study were mutually exclusive and are presented under study Part 2 in Section 3.

### 2.2 Recruitment and inclusion/exclusion criteria

Nulliparous women with singleton pregnancies attending antenatal clinics at the Adelaide, South Australia and Auckland, New Zealand SCOPE centres between November 2004 and September 2008, at 15 ± 1 weeks of gestation, were recruited and provided written informed consent. Women at high risk for pregnancy complications because of underlying medical, gynaecological history, three or more miscarriages or terminations of pregnancy were not eligible. Partners were invited to participate and provided written informed consent. Participants were excluded after recruitment due to pregnancy loss, protocol violation or loss to follow‐up.

### 2.3 Data collection

Participants were interviewed and examined by research midwives at 15 ± 1 and 20 ± 1 weeks of gestation. Data collection included demographic information with socio‐economic index (SEI) classified using New Zealand criteria (scale of 10–90 with 10 representing the lowest SEI) (SNZ, 2003), medical history, obstetric and family history, nutrient supplementation, diet, smoking, alcohol and the use of recreational drugs. Supplementation was recorded as total daily dose of FA. FA supplementation dose was calculated from the strength and quantity of tablets consumed and included both discrete FA tablets and FA contained in multivitamin preparations. Supplementation dose of 800‐μg daily FA was used as a reference dose because in Australia, Elevit (Bayer) is the most popular brand of FA supplementation containing 800 μg of FA. Maternal height, weight and blood pressure were obtained at 15 ± 1 weeks of gestation. Umbilical and uterine artery Doppler studies were performed at 20‐week gestation by an experienced sonographer as previously described (Groom et al., 2009) and data used to calculate the umbilical and uterine artery resistance indices (RI). Abnormal umbilical and uterine artery indices were defined as >90th centile. All women were followed prospectively, and pregnancy outcome data and infant measurements were recorded within 72 h of birth.

### 2.4 Sample collection

Peripheral blood samples were collected, placed immediately on ice, processed and stored at −80°C within 3 h. DNA was extracted from buffy coats from EDTA peripheral blood samples (QIAamp 96 DNA blood kit, QIAGEN, USA) collected from all women and most partners. Saliva samples were collected from partners who were unwilling to undergo venipuncture, using Oragene kits (DNA Genotek Inc., USA).

### 2.5 Genotyping

Genotyping data were available for 2,842 (89%) of the 3,196 women. Genotyping for *MTHFR C677T* rs1801133, *MTHFR A1298C* rs1801131, *MTHFD1 G1958A* rs2236225, *MTR A2756G* rs1805087, *MTRR A66G* rs1801394 and *TCN2 C766G* rs1801198 single nucleotide polymorphisms (SNPs) was performed at the Australian Genome Research Facility (Brisbane) using the Sequenom MassARRAY system (Agena Bioscience, USA). For quality control, each sample was also genotyped for amelogenin to ensure that the sex of the sample was correct (Sullivan, Mannucci, Kimpton, & Gill, 1993). Chi‐squared test was used to test genotypes for Hardy–Weinberg Equilibrium.

### 2.6 Dietary intake

Aspects of maternal preconception (1 month prior to becoming pregnant) and early pregnancy (up until the first antenatal visit) dietary intake were recorded by the research midwife at 15 ± 1 weeks of gestation. Fast food consumption was defined as the combined number of serves of burgers, fried chicken, pizza and fried hot chips per week and was categorised as low (≤2 serves per week), moderate (>2 and ≤4 serves per week) and high (>4 serves per week) (Grieger et al., 2018). Fruit and green leafy vegetable consumption was categorised as low (<1 serve per day) or moderate (1 or more serves per day). Green leafy vegetables category included all brassicas.

### 2.7 Biochemical measurements

Complete biochemistry data were only available for 325 (10%) women from the Adelaide SCOPE cohort. Plasma and serum sampled at 15 ± 1 weeks of gestation were used to measure circulating folate (nmol L^−1^), vitamin B12 (pmol L^−1^) and Hcy (μmol L^−1^). Serum folate and vitamin B12 were quantified using the ARCHITECT® folate and B12 assays (Abbott Laboratories, Abbott Park, IL, USA). Total L‐Hcy in plasma was quantified using the AxSYM® homocysteine assay (Abbott, Wiedbaden, Germany). Quantification was performed by the Division of Clinical Biochemistry and Immunology, SA Pathology, Adelaide.

### 2.8 Definitions of pregnancy outcomes

GHT was defined as blood pressure ≥140/90 mmHg arising after 20‐week gestation without any other feature of the multisystem disorder PE that resolves within 3‐month postpartum (Brown et al., 2000). PE was defined as GHT with proteinuria (24‐h urinary protein level of >300 mg or a spot urine protein: creatinine ratio of ≥30 mg mmol^−1^ creatinine or urine dipstick protein level ≥ ++) or any multisystem complication of PE, including fetal growth restriction (WHO recommendations, 2018). SGA was defined as birth weight below the 10th customised centile adjusted for maternal height, weight, parity, ethnicity, gestational age at delivery and infant sex (McCowan, Stewart, Francis, & Gardosi, 2004). sPTB was spontaneous preterm labour or preterm premature rupture of the membranes resulting in birth at <37‐week gestation. GDM was defined as glucose intolerance that arises during pregnancy and is diagnosed around 26‐week gestation by glucose challenge test using the new (2016) World Health Organisation classification (fasting glucose of ≥5.1 mmol L^−1^ or following an Oral Glucose Tolerance Test, a 2‐h level of ≥8.5 mmol L^−1^). Uncomplicated pregnancy was defined as a pregnancy in which no antenatal medical or obstetric complication had been diagnosed, resulting in delivery of an appropriately grown, healthy baby at ≥37 weeks of gestation.

### 2.9 Statistics

All data analyses were performed using R version 3.5.3. Results were reported as number and percent (*n* [%]) or mean ± standard deviation (SD) where appropriate. Multivariable logistic regression was used to determine the odds (with corresponding 95% confidence intervals) of known SNPs in genes involved in the one‐carbon metabolic pathway for all five pregnancy complications, PE, GHT, SGA, sPTB and GDM, adjusted for maternal age, body mass index (BMI), smoking status and study centre. The relationships between SNPs and folate, vitamin B12 and Hcy were also examined using linear regression, with maternal age, BMI, smoking status and FA supplementation at 15 ± 1 weeks of gestation as covariates. Descriptive statistics were reported for maternal age, SEI, BMI and smoking status according to FA supplementation status at 15‐week gestation and for blood biochemistry measures (folate, vitamin B12 and Hcy) within the Adelaide 325 cohort where data were available. The association between FA supplementation and neonatal outcomes was further examined using linear regression adjusted for maternal age, BMI, smoking status and study centre. Further analysis on folate, vitamin B12 and Hcy, available within the Adelaide 325 cohort, was performed using linear and logistic regression to examine their relationship with pregnancy outcomes, adjusting for maternal age, BMI and smoking status. Blood biochemistry results, including the effects of folate:vitaminB12 ratio, on pregnancy outcomes were presented as odds ratios (95% CI). For testing an effect (RR) of 1.5 with a sample size of 325, assuming a standard deviation of 10 (SD of serum folate from 325 data), the power is 77.8%.

To examine the association between dietary intake (at preconception and 15‐week gestation) and pregnancy outcomes, logistic regression was performed adjusting for maternal age, BMI, smoking status, FA supplementation and study centre. Odds ratios (95% CI) were reported. Similarly, linear regression was performed for continuous neonatal outcomes and maternal blood biochemistry. *P* < .05 was considered statistically significant.

### 2.10 Ethical considerations

Ethical approval was obtained in Adelaide from the Central Northern Adelaide Health Service Human Research Ethics Committee (REC 1712/5/2008) and in Auckland by the Northern Region Ethics Committee (AKX/02/00/364). The SCOPE study was registered with the Australian New Zealand Clinical Trial Registry (ACTRN12607000551493).

## 3 RESULTS

### 3.1 Adelaide and Auckland SCOPE 3196 cohort

#### 3.1.1 Baseline characteristics

The baseline characteristics data for SCOPE women included in this study are summarised in Table 1. The mean age of women in the study was 28 years. Women who developed PE were younger, had lower SEI, higher BMI and mean arterial blood pressure (MAP) at 15‐week gestation and gave birth to babies of lower birth weight and gestational age compared with women without PE. Women who developed GHT also had lower SEI and higher BMI and MAP at 15‐week gestation compared with women without GHT; however, these women gave birth to babies of higher birth weight. Women who delivered a SGA baby had higher MAP at 15‐week gestation, and their babies were of lower birth weight and gestational age compared with non‐SGA cases. Women who developed GDM had a higher BMI and MAP at 15 ± 1 weeks of gestation and, as per clinical management strategy, gave birth to babies of lower gestational age compared with women without GDM.

**TABLE 1:** Maternal characteristics and pregnancy data at 15 ± 1 weeks of gestation and neonatal birth data according to pregnancy outcome

	Non‐PE *n* = 2,962	PE *n* = 222	*P*	Non‐GHT *n* = 2,994	GHT *n* = 188	*P*	Non‐SGA *n* = 2,882	SGA *n* = 298	*P*	Non‐sPTB *n* = 3,028	sPTB *n* = 156	*P*	Non‐GDM *n* = 2,869	GDM *n* = 104	*P*
**Maternal age (years)**	28.1 ± 5.8	26.9 ± 5.9	***.009***	28.0 ± 5.8	27.6 ± 6.1	.401	27.9 ± 5.8	28.4 ± 6.0	.240	27.9 ± 5.8	27.7 ± 6.2	.616	27.9 ± 5.9	28.9 ± 5.2	.080
**SEI**	40.8 ± 16.5	37.8 ± 16.5	***.008***	40.8 ± 16.4	37.6 ± 16.9	***.010***	40.7 ± 16.5	39.5 ± 16.1	.204	40.6 ± 16.5	39.8 ± 16.4	.558	40.5 ± 16.6	37.9 ± 16.4	.114
**MAP**	78.5 ± 7.9	83.9 ± 8.5	***<.0001***	78.5 ± 7.9	84.9 ± 8.0	***<.0001***	78.8 ± 8.0	79.8 ± 8.5	***.036***	78.8 ± 8.0	79.4 ± 8.3	.355	78.7 ± 7.9	84.1 ± 9.8	***<.0001***
**BMI**	25.4 ± 5.1	28.5 ± 7.2	***<.0001***	25.4 ± 5.2	28.8 ± 6.1	***<.0001***	25.6 ± 5.3	25.7 ± 5.3	.866	25.6 ± 5.3	25.7 ± 5.7	.731	25.5 ± 5.2	30.1 ± 6.8	***<.0001***
**Folic acid supplementation**	2,212 (74.7%)	173 (77.9%)	.298	2,240 (74.8%)	145 (77.1%)	.544	2,175 (75.5%)	208 (69.8%)	***.035***	2,273 (75.1%)	112 (71.8%)	.345	2,160 (75.3%)	81 (77.9%)	.643
**Smokers:**															
Never	2,261 (76.3%)	166 (74.8%)	Ref	2,289 (76.5%)	138 (73.4%)	Ref	2,214 (76.8%)	211 (70.8%)	Ref	2,319 (76.6%)	108 (69.2%)	Ref	2,185 (76.2%)	81 (77.9%)	Ref
Quit before pregnancy	53 (1.8%)	6 (2.7%)	.323	52 (1.7%)	7 (3.7%)	.051	58 (2%)	1 (0.3%)	.091	57 (1.9%)	2 (1.3%)	.698	51 (1.8%)	2 (1.9%)	.938
Quit before 15 weeks	317 (10.7%)	26 (11.7%)	.613	320 (10.7%)	22 (11.7%)	.580	316 (11%)	26 (8.7%)	.497	328 (10.8%)	15 (9.6%)	.949	313 (10.9%)	10 (9.6%)	.663
Smoking at 15 weeks	331 (11.2%)	24 (10.8%)	.956	333 (11.1%)	21 (11.2%)	.852	294 (10.2%)	60 (20.1%)	***<.0001***	324 (10.7%)	31 (19.9%)	***.001***	320 (11.2%)	11 (10.6%)	.817
Uterine a. RI	0.56 ± 0.10	0.60 ± 0.11	<.0001	0.56 ± 0.10	0.56 ± 0.10	.482	0.56 ± 0.10	0.61 ± 0.10	***<.0001***	0.56 ± 0.10	0.59 ± 0.11	***.005***	0.56 ± 0.10	0.57 ± 0.10	.665
Umbilical RI	0.73 ± 0.07	0.74 ± 0.07	***.019***	0.73 ± 0.07	0.73 ± 0.06	.245	0.73 ± 0.07	0.74 ± 0.07	***.002***	0.73 ± 0.07	0.72 ± 0.06	.170	0.73 ± 0.07	0.74 ± 0.07	.387
Birth weight (g)	3420.3 ± 584.6	2954.6 ± 741.3	***<.0001***	3380.8 ± 615.7	3499.8 ± 466.0	.201	3477.1 ± 528.6	2546.4 ± 629.4	***<.0001***	3439.2 ± 553.7	2378.4 ± 743.2	***<.0001***	3,413 ± 545.2	3311.9 ± 644.9	.065
Gestational age (weeks)	39.6 ± 2.4	38.2 ± 2.5	***<.0001***	39.5 ± 2.4	39.6 ± 1.5	.737	39.6 ± 2.0	38.5 ± 4.1	***<.0001***	39.7 ± 1.9	33.8 ± 3.9	***<.0001***	39.6 ± 1.7	38.5 ± 2.2	***<.0001***

*Note*: Customised birth weight centiles indicate fetal weight according to maternal height, weight, ethnicity, parity, gestation and sex of the baby. All data are represented as mean ± SD or number of women (%); *P* values in bold are statistically significant.

Abbreviations: BMI, body mass index; MAP, mean arterial pressure; SEI, socio‐economic index; uterine a RI, uterine artery resistance index.

There was no association between smoking at 15‐week gestation and PE, GHT or GDM. However, more women with SGA and sPTB smoked cigarettes at 15 ± 1 weeks of gestation (SGA 20.1% vs. non‐SGA 10.1%; *P* < .0001 and sPTB 19.9% vs. non‐sPTB10.7%; *P* = .001) (Table 1).

#### 3.1.2 Maternal and paternal SNPs and pregnancy outcomes

The maternal genotype rs1801131 *MTHFR A1298C* (CC) was associated with increased risk for PE (OR 1.80, 1.13–2.88) when compared with the AA genotype, whereas rs1801198 *TCN2 C677G* (GG) was associated with a decreased risk for sPTB (OR 0.60, 0.36–0.99) when compared with CC genotype (Table S1). The paternal genotypes rs1801131 *MTHFR A1298C* (CC) and rs2236225 *MTHFD1 G1958A* (AA) were associated with a decreased risk for sPTB (OR 0.32, 0.12–0.89 and OR 0.52, 0.28–0.99, respectively), whereas the paternal *MTHFR C677T* (CT) genotype was associated with an increased risk for GHT (OR 1.60, 1.08–2.39). Testing for compound heterozygous *MTHFR* (*C677T* and *A1298C*) revealed no further associations with any of the pregnancy outcomes measured.

#### 3.1.3 FA supplementation

Approximately 55% of the Adelaide and Auckland SCOPE women included in this study took supplements containing FA preconception, which increased to 75% of women at 15 ± 1 weeks of gestation. Women who supplemented with <800‐μg daily FA at 15 ± 1 weeks of gestation were younger (26.2 ± 5.9 years vs. 27.1 ± 6.3 years; mean ± SD) and had lower SEI (34.7 ± 15.4 vs. 40.0 ± 16.4; mean ± SD) compared with women who did not use FA supplementation. Conversely, women who supplemented with ≥800‐μg FA at 15 ± 1 weeks of gestation were older (29.6 ± 5.1 years vs. 27.1 ± 6.3 years; mean ± SD) and had higher SEI (44.9 ± 15.9 vs. 40.0 ± 16.4; mean ± SD) compared with women who did not supplement (Table 2A).

**TABLE 2:** Folic acid supplementation at 15 ± 1 weeks of gestation in association with maternal characteristics and pregnancy data, neonatal birth data and pregnancy outcomes

Table 2A					
Maternal factors	Not supplemented (*n* = 800)	<800 μg (*n* = 960)	*P*	≥800 μg (*n* = 1,436)	*P*
Maternal age (years)	27.12 ± 6.25	26.23 ± 5.87	*<.0001*	29.62 ± 5.12	*<.0001*
Socio‐economic index (SEI)	40.01 ± 16.40	34.66 ± 15.43	*<.0001*	44.86 ± 15.89	*<.0001*
BMI	25.61 ± 5.52	26.18 ± 5.54	.249	25.22 ± 4.98	.100
Smoking:					
Never	575 (71.9%)	648 (67.5%)	Ref	1,211 (84.3%)	Ref
Quit before pregnancy	16 (2%)	20 (2.1%)	.754	23 (1.6%)	.230
Quit before 15 weeks	90 (11.2%)	133 (13.9%)	.054	123 (8.6%)	*.008*
Smoking at 15 weeks	119 (14.88%)	159 (16.56%)	.191	79 (5.5%)	*<.0001*

Table 2B					
Pregnancy and neonatal data	Not supplemented (*n* = 800)	<800 μg (*n* = 960)	*P*	≥800 μg (*n* = 1,436)	*P*
		Effects (95% CI)^a^		Effects (95% CI)^a^	
Mean uterine artery RI	Reference	−0.008 (−0.017 to 0.001)	.099	−0.009 (−0.017 to −0.001)	*.037*
Mean umbilical artery RI	‐	−0.005 (−0.011 to 0.002)	.138	−0.005 (−0.011 to 0.001)	.091
Uterine artery RI > 90th^b^	‐	0.78 (0.57–1.06)	.107	0.74 (0.54–0.99)	*.047*
Umbilical artery RI > 90th^b^	‐	1.02 (0.79–1.32)	.864	0.90 (0.69–1.18)	.438
Birth weight (g)^c^	‐	35.01 (−6.35 to 76.36)	.097	40.85 (3.74–77.95)	*.031*
Gestational age (weeks)	‐	0.045 (−0.222 to 0.312)	.741	−0.043 (−0.283 to 0.196)	.724

Table 2C															
Folic acid supplementation at 15 weeks	Non‐PE	PE	%PE	Non‐GHT	GHT	%GHT	Non‐SGA	SGA	%SGA	Non‐sPTB	sPTB	%sPTB	Non‐GDM	GDM	%GDM
Not supplemented	750 (25.3%)	49 (22.1%)	6.1%	754 (25.2%)	43 (22.9%)	5.4%	707 (24.5%)	90 (30.2%)	11.3%	755 (24.9%)	44 (28.2%)	5.5%	709 (24.7%)	23 (22.1%)	3.1%
<800 μg	861 (29.1%)	96 (43.2%)	10.3%	892 (29.8%)	65 (34.6%)	6.8%	868 (30.1%)	89 (29.9%)	9.3%	911 (30.1%)	46 (29.5%)	4.8%	882 (30.7%)	31 (29.8%)	3.4%
≥800 μg	1,351 (45.6%)	77 (34.7%)	5.4%	1,348 (45%)	80 (42.6%)	5.6%	1,307 (45.4%)	119 (39.9%)	8.3%	1,362 (45%)	66 (42.3%)	4.6%	1,278 (44.5%)	50 (48.1%)	3.8%
*P* value		*.0000*			.3750			.0730			.6420			.7470	

*Note*: All data are represented as mean ± SD; *P* values in bold are statistically significant.

Abbreviations: BMI, body mass index; GDM, gestational diabetes mellitus; GHT, gestational hypertension; PE, preeclampsia; PTB, preterm birth; RI, resistance index; SGA, small for gestational age; sPTB, spontaneous preterm birth.

^a^ Effects are difference in means from Linear regression adjusted for maternal BMI, smoking status and study centre.

^b^ Effects are odds ratios from Logistic regression adjusted for maternal BMI, smoking status and study centre.

^c^ Adjusted for BMI, smoking status, gestational age and study centre.

The percentage of women who smoked at 15 ± 1 weeks of gestation did not differ amongst those who supplemented with <800‐μg daily FA at 15 ± 1 weeks of gestation and those who did not supplement (16.6% vs. 14.8%, *P* = .51). However, fewer women who supplemented with ≥800‐μg daily FA at 15 ± 1 weeks of gestation smoked in early pregnancy or at 15 weeks of gestation compared with those who did not supplement (8.6% vs. 11.2%, *P* = .008 and 5.5% vs. 14.8%; *P* < .0001, respectively; Table 2A).

Women who supplemented with ≥800‐μg FA daily at 15 ± 1 weeks of gestation had lower uterine artery RI and gave birth to babies of increased birth weight compared with women not supplementing (Table 2A).

A total of 10.3% of women who supplemented with <800‐μg daily FA at 15‐week gestation developed PE compared with 6.1% of women who did not supplement (*P* = .0096). Supplementation with ≥800‐μg daily FA at 15 ± 1 weeks of gestation was associated with a lower rate of PE (5.4%) compared with supplementation with <800 μg (10.3%; *P* = .0001; Table 2C).

#### 3.1.4 Maternal dietary intake and pregnancy health and outcomes

Women who consumed 2–4 serves of fast food per week 1 month prior to conception were more likely to develop GDM compared with women who consumed less than 2 serves per week (OR 2.45; 95% CI 1.06–5.69; Table 3), and these effects were independent of BMI and smoking.

**TABLE 3:** Maternal dietary intake preconception and at 15 ± 1 weeks of gestation and pregnancy outcome in the Adelaide and Auckland 3,196 cohort

Dietary intake	Non‐PE	PE	OR (95% CI)	Non‐GHT	GHT	OR (95% CI)	Non‐SGA	SGA	OR (95% CI)	Non‐sPTB	sPTB	OR (95% CI)	Non‐GDM	GDM	OR (95% CI)
**Fast food—PC (serves/week)**															
0 to ≤2	458 (24.9%)	19 (13.4%)	1	463 (24.9%)	14 (12.2%)	1	433 (24.1%)	43 (24.3%)	1	454 (24.2%)	23 (22.8%)	1	443 (24.5%)	7 (9.6%)	1
>2 to ≤4	870 (47.4%)	71 (50%)	1.45 (0.84–2.50)	878 (47.2%)	62 (53.9%)	1.62 (0.87–3.01)	865 (48.1%)	74 (41.8%)	0.88 (0.58–1.33)	895 (47.7%)	46 (45.5%)	0.90 (0.52–1.54)	848 (46.9%)	44 (60.3%)	***2.45 (1.06–5.69)***
>4	508 (27.7%)	52 (36.6%)	1.78 (0.96–3.32)	521 (28%)	39 (33.9%)	1.51 (0.75–3.07)	500 (27.8%)	60 (33.9%)	1.17 (0.70–1.95)	528 (28.1%)	32 (31.7%)	0.87 (0.45–1.69)	518 (28.6%)	22 (30.1%)	2.33 (0.90–6.04)
**Green vegetables—PC (serves/day)**															
<1	1,485 (50.1%)	136 (61.3%)	1	1,514 (50.6%)	105 (55.9%)	1	1,446 (50.2%)	173 (58.1%)	1	1,534 (50.7%)	87 (55.8%)	1	1,470 (51.2%)	67 (64.4%)	1
≥1	1,477 (49.9%)	86 (38.7%)	0.80 (0.59–1.08)	1,480 (49.4%)	83 (44.1%)	0.98 (0.71–1.36)	1,436 (49.8%)	125 (41.9%)	***0.73 (0.56–0.95)***	1,494 (49.3%)	69 (44.2%)	0.91 (0.64–1.30)	1,399 (48.8%)	37 (35.6%)	***0.63 (0.40–0.99)***
**Fruit—PC (serves/day)**															
<1	1,140 (38.5%)	108 (48.6%)	1	1,162 (38.8%)	85 (45.2%)	1	1,106 (38.4%)	141 (47.3%)	1	1,168 (38.6%)	80 (51.3%)	1	1,140 (39.7%)	48 (46.2%)	1
≥1	1822 (61.5%)	114 (51.4%)	0.86 (0.63–1.18)	1832 (61.2%)	103 (54.8%)	0.97 (0.69–1.36)	1776 (61.6%)	157 (52.7%)	***0.72 (0.54–0.94)***	1860 (61.4%)	76 (48.7%)	***0.66 (0.46–0.95)***	1729 (60.3%)	56 (53.8%)	0.93 (0.60–1.46)
**Fast food—15 weeks (serves/week)**															
0 to ≤2	517 (28.1%)	29 (20.4%)	1	528 (28.3%)	18 (15.7%)	1	491 (27.2%)	53 (29.9%)	1	517 (27.5%)	29 (28.7%)	1	499 (27.5%)	16 (21.9%)	1
>2 to ≤4	933 (50.7%)	75 (52.8%)	1.06 (0.67–1.69)	939 (50.3%)	68 (59.1%)	1.56 (0.90–2.70)	930 (51.6%)	77 (43.5%)	0.78 (0.53–1.14)	955 (50.8%)	53 (52.5%)	0.88 (0.54–1.43)	922 (50.9%)	40 (54.8%)	0.95 (0.50–1.77)
>4	390 (21.2%)	38 (26.8%)	1.28 (0.74–2.22)	399 (21.4%)	29 (25.2%)	1.42 (0.74–2.72)	381 (21.1%)	47 (26.6%)	1.03 (0.64–1.66)	409 (21.7%)	19 (18.8%)	0.59 (0.31–1.13)	392 (21.6%)	17 (23.3%)	1.00 (0.46–2.17)
**Green vegetable—15 weeks (serves/day)**															
<1	1,592 (53.7%)	146 (65.8%)	1	1,626 (54.3%)	110 (58.5%)	1	1,555 (54%)	181 (60.7%)	1	1,652 (54.6%)	86 (55.1%)	1	1,583 (55.2%)	62 (59.6%)	1
≥1	1,370 (46.3%)	76 (34.2%)	0.74 (0.55–1.01)	1,368 (45.7%)	78 (41.5%)	0.99 (0.73–1.38)	1,327 (46%)	117 (39.3%)	***0.77 (0.60–0.99)***	1,376 (45.4%)	70 (44.9%)	1.09 (0.78–1.54)	1,286 (44.8%)	42 (40.4%)	0.95 (0.62–1.46)
**Fruit—15 weeks (serves/day)**															
<1	742 (25.1%)	75 (33.8%)	1	753 (25.2%)	63 (33.5%)	1	725 (25.2%)	91 (30.5%)	1	761 (25.1%)	56 (35.9%)	1	743 (25.9%)	29 (27.9%)	1
≥1	2,220 (74.9%)	147 (66.2%)	0.91 (0.66–1.27)	2,241 (74.8%)	125 (66.5%)	0.84 (0.59–1.20)	2,157 (74.8%)	207 (69.5%)	0.78 (0.58–1.05)	2,267 (74.9%)	100 (64%)	***0.65 (0.44–0.96)***	2,126 (74.1%)	75 (72.1%)	1.17 (0.72–1.91)

*Note*: *P* values in bold/italics are statistically significant. Data are corrected for maternal age, BMI, smoking, folate supplement and study centre.

Abbreviations: CI, confidence interval; GDM, gestational diabetes mellitus; GHT, gestational hypertension; OR, odds ratio; PC, preconception; PE, preeclampsia; PTB, preterm birth; SGA, small for gestational age; sPTB, spontaneous preterm birth.

Women who consumed one or more serves of green vegetables per day, 1 month prior to conception and at 15‐week gestation, had reduced odds of having a SGA baby compared with women who consumed less than 1 serve of green vegetables per day (OR 0.73; 95% CI 0.57–0.94 and OR 0.74; 95% CI 0.58–0.94, respectively; Table 3). Green vegetables consumption prior to pregnancy was also associated with a reduced risk of having GDM (OR 0.63; 95% CI 0.40–0.99), with fewer women who developed GDM consuming one or more serves of green vegetables per day compared with women without GDM (35.6% vs. 48.8%).

Women who consumed one or more serves of fruit per day prior to conception had reduced odds of SGA (OR of 0.75; 95% CI 0.58–0.97) and sPTB (OR 0.66; 95% CI 0.46–0.95) (Table 3). Fruit consumption at 15‐week gestation was also associated with a reduced odds of having sPTB (OR 0.65; 95% CI 0.44–0.96), with fewer women with sPTB consuming one or more serves of fruit per day at 15 weeks compared with women without sPTB (64% vs. 74.9%).

There was no association between maternal diet and gestational age at delivery or between maternal diet and mean birth weight when birth weight was adjusted for maternal age, BMI, smoking, FA supplementation, study centre and gestational age (Table S2).

### 3.2 Adelaide nested case–control 325 cohort study

#### 3.2.1 FA supplementation and blood biochemistry

In a subset of 325 women from the Adelaide SCOPE cohort for whom blood biochemistry data were available, we assessed the relationship between FA supplementation and circulating serum folate, serum vitamin B12 and plasma Hcy levels at 15 ± 1 weeks of gestation. In this subset, 30% of women supplemented with FA preconception, which increased to 80% at 15 ± 1 weeks of gestation. Unsurprisingly, women who supplemented with either <800‐ or ≥800‐μg daily FA at 15 ± 1 weeks of gestation had increased circulating folate and vitamin B12 levels compared with those who did not supplement. Mean circulating folate concentration in women who did not supplement with FA was 24.0 ± 10.3 nmol L^−1^ (mean ± SD), and it increased to 33.9 ± 9.1 nmol L^−1^ (in women who supplemented with <800‐μg daily FA; *P* < .0001) and 38.9 ± 6.8 nmol L^−1^ (in women who supplemented with ≥800‐μg daily FA; *P* < .0001). Mean circulating vitamin B12 concentration in women who did not supplement was 248.2 ± 76.5 pmol L^−1^ (mean ± SD), and it increased to 282.9 ± 101.8 pmol L^−1^ (in women who supplemented with <800‐μg daily FA; *P* = .023) and 289.5 ± 126.8 pmol L^−1^ (in women who supplemented with ≥800‐μg daily FA; *P* = .019). Women who supplemented with ≥800‐μg daily FA had lower Hcy levels (6.1 ± 1.1 μmol L^−1^; mean ± SD) compared with those who did not supplement (6.4 ± 1.4 μmol L^−1^); however, this difference was not statistically significant (*P* = .074).

Hcy was however negatively correlated with serum folate and vitamin B12 levels (*r* = −0.262, *P* < .001 and *r* = −0.283, *P* < .001, respectively).

#### 3.2.2 Blood biochemistry in association with SNPs and maternal lifestyle factors

A borderline increase in circulating Hcy levels was measured in women with rs2236225 *MTHFD1* GA and AA genotypes compared with those with GG genotype (Hcy mean ± SD: GG = 6.0 ± 1.3 μmol L^−1^; GA = 6.4 ± 1.2 μmol L^−1^; AA=6.4±1.5 μmol L^−1^; *P* = 2.046), when data were corrected for maternal age, BMI, smoking and FA supplementation. Although women with rs1801133 *MTHFR C677T* (TT) genotype had increased circulating Hcy (mean ± SD: 6.5±1.1 μmol L^−1^) and reduced serum folate (mean ± SD: 30.5±11.5 nmol L^−1^) compared with those with CC genotype (mean ± SD: 6.2±1.2 μmol L^−1^ and 34.1±10.5 nmol L^−1^, respectively), these differences were not statistically significant (*P* = .219), possibly due to the small number of women with the TT genotype (TT *n* = 38; CC *n* = 149). There was no association between maternal *MTR*, *MTRR* and *TCN2* genotypes and plasma Hcy concentration. The outcomes were the same when data were adjusted for folate and vitamin B12.

Hcy levels were higher in women who smoked at 15 ± 1 weeks of gestation compared with nonsmokers (8.2% increase [95% CI 2.7–14.1%]; *P* = .003), whereas serum folate and vitamin B12 levels were lower (28.2% decrease in folate [95% CI 20.3–35.3%]; *P* < .0001; and 10.5% decrease in vitamin B12 [95% CI 1.7–18.5%]; *P* = .021) in smokers compared with nonsmokers. Circulating folate, vitamin B12 and Hcy levels were not associated with alcohol consumption, but this could be a reflection of low number of women who consumed alcohol (*n* = 13) compared with those who did not (*n* = 312); mean folate, vitamin B12 and Hcy levels in nonconsumers versus consumers of alcohol were 33.3 ± 10.1 vs. 32.3 ± 10.7 nmol L^−1^, *P* = .734; 277.8 ± 105.2 vs. 268.2 ± 95.7 pmol L^−1^, *P* = .748; 6.2 ± 1.3 vs. 6.2 ± 1.4 μmol L^−1^, *P* = .858, respectively. Maternal age was associated with increased serum folate (1.015 [1.006–1.023]), whereas maternal MAP and BMI were associated with lower serum vitamin B12 (0.995 [0.990–0.999] and 0.990 [0.985–0.996], respectively), presented as effects (95% CI), which are ratios of geometric means.

There was no association between fast food or fruit consumption and blood biochemistry. However, women who, at 15‐week gestation, consumed one or more serves of green leafy vegetables per day had higher circulating vitamin B12 levels than those who consumed less than one serve per day (298.5 ± 14.7 vs. 271 ± 6 pmol L^−1^; *P* = .048). This relationship was strengthened when corrected for maternal age, BMI, smoking and FA supplementation (*P* = .037; Table S3).

#### 3.2.3 Blood biochemistry in association with pregnancy outcomes

Serum folate levels were higher in women who developed GHT (36.7 ± 7.1 nmol L^−1^) and GDM (37.6 ± 8 nmol L^−1^) compared with those with uncomplicated pregnancies (31.9 ± 11.2 nmol L^−1^; *P* = .005 and *P* = .007, respectively), but the effect of serum folate on the risk of developing GDM was lost after adjusting for potential confounders (Table 4A).

**TABLE 4:** Maternal blood biochemistry data at 15 ± 1 weeks of gestation and pregnancy and neonatal outcomes in the nested case–control study

(A) Effect (95% CI) of circulating folate, vitamin B12 and Hcy on pregnancy outcome											
	Uncomplicated (*N* = 111)	PE (*N* = 48)	*P*	GHT (*N* = 46)	*P*	SGA (*N* = 44)	*P*	sPTB (*N* = 43)	*P*	GDM (*N* = 33)	*P*
		OR (95% CI)		OR (95% CI)		OR (95% CI)		OR (95% CI)		OR (95% CI)	
Folate^a^ (nmol L^−1^)	Reference	1.05 (0.86–1.27)	.644	1.36 (1.10–1.68)	*.004*	1.11 (0.92–1.34)	.275	0.90 (0.75–1.09)	.279	1.22 (0.93–1.59)	.149
Vitamin B12^b^ (pmol L^−1^)	Reference	0.89 (0.72–1.09)	.262	0.94 (0.80–1.11)	.487	0.88 (0.73–1.06)	.172	0.84 (0.69–1.02)	.085	0.99 (0.83–1.18)	.913
Hcy^c^ (μmol L^−1^)	Reference	1.15 (0.86–1.53)	.361	1.05 (0.77–1.42)	.761	1.24 (0.92–1.68)	.153	1.02 (0.75–1.38)	.906	0.82 (0.55–1.23)	.333
Folate:B12 ratio^d^	Reference	1.24 (0.67–2.29)	.496	1.98 (1.13–3.47)	*.017*	1.72 (0.91–3.25)	.093	1.17 (0.65–2.11)	.607	1.37 (0.71–2.62)	.347

(B) Effect (95% CI) of circulating folate, vitamin B12 and Hcy on pregnancy and neonatal data						
	Uterine RI	Umbilical RI	Uterine RI >90th^e^	Umbilical RI >90th^e^	Birth weight (g)	Gestational age
Folate^a^ (nmol L^−1^)	−0.003 (−0.007 to 0.002)	−0.001 (−0.005 to 0.002)	0.966 (0.820*–*1.148)	0.918 (0.801*–*1.057)	34.579 (−1.747 to 70.904)	0.013 (−0.106 to 0.131)
B12^b^ (pmol L^−1^)	−0.004 (−0.009 to 0.001)	0.000 (−0.003 to 0.003)	0.925 (0.762*–*1.094)	1.040 (0.905*–*1.181)	18.195 (−17.142 to 53.533)	0.052 (−0.077 to 0.181)
Hcy^c^ (μmol L^−1^)	0.004 (−0.003 to 0.012)	*0.006 (0.001–0.011)*	1.092 (0.835*–*1.409)	1.061 (0.845*–*1.320)	−56.891 (−114.495 to 0.712)	0.018 (−0.164 to 0.200)
Folate:B12 ratio^d^	0.003 (−0.013 to 0.018)	−0.001 (−0.011 to 0.009)	1.074 (0.627*–*1.838)	0.792 (0.486*–*1.289)	−6.615 (−118.339 to 105.110)	−0.2 (−0.571 to 0.172)

*Note*: Data are adjusted for maternal age, BMI and smoking status at 15 weeks. *P* values in bold are statistically significant.

Abbreviations: CI, confidence interval; GDM, gestational diabetes mellitus; GHT, gestational hypertension; OR, odds ratio; PE, preeclampsia; RI, resistance index; SGA, small for gestational age; sPTB, spontaneous preterm birth.

^a^ Effect for every 5‐unit increase in folate.

^b^ Effect for every 50‐unit increase in B12.

^c^ Effect for every 1 unit increase in Hcy.

^d^ Effect for every 0.1 unit increase in folate: B12 ratio.

^e^ Odds ratios estimated from Logistic Regression.

Serum vitamin B12 levels were not associated with any of the five adverse pregnancy outcomes assessed.

Compared with women who had an uncomplicated pregnancy, women who developed GHT had higher serum folate to vitamin B12 ratio at 15‐week gestation (1.98; 95% CI: 1.13–3.47; *P* = .017).

While plasma Hcy at 15 ± 1 weeks of gestation was increased in women who delivered an SGA baby compared with those with uncomplicated pregnancies (6.6 ± 0.2 μmol L^−1^ compared with 6.1 ± 0.1 μmol L^−1^), the significance of the association was lost after adjusting for potential confounders. Higher circulating Hcy levels were positively associated with umbilical RI (1 μmol L^−1^ increase is associated with a 0.006 increase in umbilical RI; 95% CI 0.001–0.0110) (Table 4B).

## 4 DISCUSSION

Polymorphisms in genes involved in the one‐carbon metabolic pathway can alter enzyme activity and thus folate, vitamin B12 and Hcy metabolism (Fredriksen et al., 2007; Ganz et al., 2016; Guinotte et al., 2003). MTHFD1 plays an important role in the flux between 5,10‐methylene THF and 10‐formyl THF thereby influencing the availability of 5‐methyl THF for Hcy re‐methylation (Horne, 2003). *MTHFD1 G1958A* mutation has been shown to reduce the stability of its synthetase domain, altering its metabolic activity to limit the availability of methyl THF (Christensen et al., 2009). It is therefore unsurprising that there were associations between the maternal GA and AA genotype and higher circulating Hcy levels. Impaired MTHFD1 enzyme function has also been shown to affect purine synthesis and pregnancy outcome, with the AA genotype being linked with neural tube defects and cardiac malformations in humans (Christensen et al., 2009; Parle‐McDermott et al., 2006) and developmental defects in animal models (Christensen et al., 2013). Paternal *MTHFD1 G1958A* (AA) genotype on the other hand and *MTHFR A1298C* (CC) genotype were both associated with reduced risk of sPTB. Previous studies have shown that low folate is associated with increased risk for sPTB; however, our results show that these two paternal genotypes that are normally associated with low folate reduced the risk of sPTB. These conflicting results may be due to the fact that the paternal genotype did not impact on the maternal levels of folate, particularly considering that majority of women recruited were supplementing during early pregnancy, which would override genetic influence.

We found a significant association between the maternal *MTHFR A1298C* SNP and PE but surprisingly no association between the maternal *MTHFR C677T* (TT) genotype and any of the pregnancy outcomes. Paternal *MTHFR C677T* (CT) genotype was however associated with GHT. This association is difficult to interpret for two reasons. Firstly, the association is detected with the heterozygous genotype, which has been shown to reduce MTHFR activity but not to the same degree as the homozygous genotype (TT). If the association is due to the impact on MTHFR activity, one would expect to also see an association with the TT genotype. Secondly, it is currently unknown if the paternal genotype can influence maternal blood pressure levels; however, it is plausible that the fetal and placental genotype could impact on maternal blood flow and blood pressure, which is indirectly linked to the paternal genotype. Furthermore, McNulty et al. (2017) have shown that riboflavin, the cofactor for MTHFR, may be associated with risk for blood pressure in association with the MTHFR genotype (McNulty, Strain, Hughes, & Ward, 2017). Future studies investigating MTHFR and hypertension in pregnancy would benefit from measuring riboflavin levels and investigating this gene–nutrient interaction. MTHFR is an enzyme involved in the conversion of 5,10‐methylenetetrahydrofolate to 5‐methyltetrahydrofolate. The former is the form of folate found mainly within cells, whereas the latter is the main form of circulating folate found in blood (Pfeiffer et al., 2015) that is used for Hcy re‐methylation. Polymorphisms in both *MTHFR A1298C* (CC) and *C677T* (TT) are reported to have decreased enzyme activity and are thus associated with impaired production of 5‐methyl THF (van der Put & Blom, 2000), which would explain the associations between the *A1298C* (CC) genotype and PE and sPTB but not the lack of association between maternal *C677T* SNP and any of the pregnancy outcomes, which could be due to only 9.8% of women having the TT genotype. The low number of women with the TT genotype could also explain the lack of statistical significance in the observed decrease in circulating folate and increase in Hcy levels in women with the *C677T* (TT) genotype. Others (Parle‐McDermott et al., 2006; van der Put & Blom, 2000) have previously suggested that the combination of the expression of two genotypes, MTHFR *A1298C* and *C677T*, is important in terms of red cell folate levels and circulating Hcy levels. However, further examination of the effects of a combination of *MTHFR A1298C* and *C677T* genotypes revealed no association with neither circulating Hcy, folate nor vitamin B12, which once again is likely due to low sample numbers in the TT genotype category.

We also found an association between the maternal *TCN2 C776G* (GG) genotype and a decreased risk for sPTB. Transcobalamin 2 (TCN2) is a protein that is essential for binding circulating vitamin B12 for its transport into cells (Oussalah, Levy, Filhine‐Tresarrieu, Namour, & Gueant, 2017). *TCN2* polymorphism affects not only its expression but ultimately its plasma concentration and thus the availability of vitamin B12 to aid in re‐methylation of Hcy (Oussalah et al., 2017). The C > G substitution alters the structure of the TCN2 protein affecting its affinity for vitamin B12, resulting in easier release from the bound TCN2 transporter (Afman, Lievers, van der Put, Trijbels, & Blom, 2002; Joslin, Green, German, & Lange, 2014) and thus reduced vitamin B12 transport into cells and ultimately higher Hcy levels compared with the CC genotype. This association conflicts with the findings from our study indicating the maternal *TCN2 C776G* (GG) genotype is associated with reduced risk for sPTB. However, because majority of women were taking a supplement containing vitamin B12, it is likely their increased B vitamin intake has outweighed the genetic influence on B12 levels. Furthermore, in this study, we have shown that BMI and maternal MAP were significantly associated with vitamin B12 levels.

Pre‐existing hypertension was an exclusion criterion for SCOPE recruitment (Andraweera et al., 2018). However, women who developed a pregnancy complication, with the exception of sPTB, had higher MAP at 15‐week gestation compared with the rest of the cohort. We found that increased umbilical artery RI at 15‐week gestation, suggesting poor placental blood supply, was associated with increased plasma Hcy. We also found that women who supplemented with FA at 15‐week gestation had reduced umbilical artery RI. As FA supplementation was associated with increased serum folate levels, these findings are consistent with evidence suggesting that high Hcy and low folate levels may be early markers for women at risk of poor placentation and subsequent pregnancy complications (Furness et al., 2013). The negative correlation between Hcy and circulating folate and vitamin B12, in the current study of low‐risk nulliparous women, is in agreement with what is currently known about Hcy and pregnancy health from our previous work (Furness et al., 2013; Furness, Yasin, Dekker, Thompson, & Roberts, 2012). However, we found no direct association between low folate levels and some pregnancy complications. Limited availability of folate results in an increased use of choline derived betaine as an alternative methyl donor for the conversion of Hcy to methionine (Ganz et al., 2016), which could explain the lack of association between serum folate levels and pregnancy outcomes in our study.

Our observation of associations between serum folate levels and GHT, as well as FA supplementation and PE, are unexpected. Although the higher incidence of PE in women who supplemented with <800‐μg daily FA at 15‐week gestation could reflect their lower SEI status, further studies are needed to determine the association between higher serum folate levels and GHT. Further studies are warranted to confirm and determine the relevance of this, given the recommended FA supplementation to reduce neural tube defects and particularly in light of the mandatory FA fortification implemented globally. It will be valuable to determine what effects mandatory fortification of the food supply with FA, in Australia (Food Standards Australia New Zealand, 2007) and in many countries globally, has on pregnancy outcomes besides decreasing the incidence of neural tube defects. A recent prospective cohort study from China reported an association between FA supplementation during pregnancy and risk for GDM (Zhu et al., 2016), which is in line with our observations on increased serum folate levels in SCOPE women who developed GDM, the latter observation warranting further investigation.

In addition to genetic and biochemical factors, this study also highlights the importance of maternal diet on pregnancy health and outcome. This information is highly relevant for all women of childbearing age particularly for those living with social and economic disadvantage, as represented here by the subset of the Adelaide cohort, where the intake of fruit and vegetables amongst young women was very low and that of fast food was very high, with close to 50% of women reporting that they consumed at least four serves of fast food each week.

We have shown that women who consumed 2–4 serves of fast food per week prior to conception had an increased risk of developing GDM compared with all other women in the cohort, which is in line with reports by others (Dominguez et al., 2014; Lamyian et al., 2017). Whereas our data did not show an association with fast food intake and Hcy levels, most likely due to the small numbers in the ‘low’ fast food intake category, others have shown an association between high‐fat diet and increased Hcy (Berstad et al., 2007). We have, however, shown that increased consumption of green leafy vegetables in early pregnancy, as well as FA supplementation prior to conception and during early pregnancy, associates with higher circulating serum vitamin B12 and folate required to maintain Hcy levels. This was expected and consistent with previous reports associating folate/FA intake and increased circulating folate (Crider, Qi, Devine, Tinker, & Berry, 2018; Looman et al., 2018; McNulty et al., 2013; Wen et al., 2016) (Ostan et al., 2018). It should be noted that SCOPE study recruitment occurred prior to mandatory fortification of the food supply with FA in Australia, so it would be interesting to investigate whether FA supplementation is still beneficial in increasing circulating folate levels in pregnant women in the current post‐fortification population.

Increased fruit and/or green vegetable consumption, as shown in this study, is also associated with reduced odds ratio for SGA (McCowan et al., 2010), sPTB and GDM. Taken together, our findings support a recommendation to increase consumption of green leafy vegetables and fruit and limit consumption of fast food, from prior to conception and in early pregnancy, in order to reduce the risks for pregnancy complications. These recommendations are in agreement with a recent report on preconception and perinatal diet and pregnancy outcome, which proposed that a Mediterranean diet comprising high protein, green leafy vegetables and fruit, reduces the risk of sPTB, whereas a high‐fat/sugar fast food diet associates with sPTB and shorter gestation (Chen et al., 2016).

A limitation of our study is the reliance on maternal recall for patient‐reported dietary consumption and a small sample size for the nested case–control study. Strengths of the study include the robust design and the collection of data using questionnaires administered at face‐to‐face interviews. Furthermore, use of a real‐time database with strict data monitoring by research midwives reduced the likelihood of data entry errors.

## 5 CONCLUSION

In summary, the main findings in this study point to an association between a combination of genetic and lifestyle factors, which reflect a period of 1 month prior to conception and early in gestation, as important parameters in determining maternal biochemistry and pregnancy health and outcome in a low‐risk nulliparous cohort. This study also highlights the importance of healthy dietary choices that include minimal consumption of fast food in combination with increased fruit and green leafy vegetable consumption prior to conception, for minimising risk for pregnancy complications. At present, there are limited studies on maternal fast food consumption, particularly prior to conception, and pregnancy outcome. Further studies are needed to assess the relevance of the association between maternal circulating folate levels and GHT, and potentially GDM, particularly in light of the global mandatory FA fortification programme implemented to reduce the incidence of neural tube defects.

## CONFLICTS OF INTEREST

The authors declare no conflicts of interest.

## CONTRIBUTIONS

TJK wrote the paper; SYL analysed the data; JAG helped with dietary analyses; PHA helped with SNPs data interpretation; DLF and DM performed the research; LEG, TBM and DCM helped with overall data analysis interpretation and provided intellectual input; CTR, GD and LMM established the research cohorts and vision; CTR designed the research study.

## Supporting information


**Table S1:** Maternal and paternal SNP genotypes and association with pregnancy outcomes
Table S2: Maternal dietary intake preconception and at 15 ± 1 weeks gestation, and birth outcomes in the Adelaide and Auckland 3,196 cohort
Table S3: Maternal dietary intake preconception and at 15±1 weeks’ gestation, and blood biochemistry in the Adelaide 325 cohort
