# Supplementary material for: Maternal folate, one‐carbon metabolism and pregnancy outcomes
Source: Matern Child Nutr. 2020 Jul 28;17(1):e13064. doi: 10.1111/mcn.13064 (PMC7729528; doi:10.1111/mcn.13064)
Supplement: Supplementary file 1 — Table S1: Maternal and paternal SNP genotypes and association with pregnancy outcomes Table S2: Maternal dietary intake preconception and at 15 ± 1 weeks gestation, and birth outcomes in the Adelaide and Auckland 3,196 cohort Table S3: Maternal dietary intake preconception and at 15±1 weeks’ gestation, and blood biochemistry in the Adelaide 325 cohort [file MCN-17-e13064-s001.docx]

**APPENDICES**

**Supplementary Table 1: Maternal and paternal SNP genotypes and association with pregnancy outcomes**

|  | **non-PE** | **PE** | **adjOR (95% CI)** | **non-GHT** | **GHT** | **adjOR (95% CI)** | **non-SGA** | **SGA** | **adjOR (95% CI)** | **non-sPTB** | **sPTB** | **adjOR (95% CI)** | **non-GDM** | **GDM** | **adjOR (95% CI)** |
| --- | --- | --- | --- | --- | --- | --- | --- | --- | --- | --- | --- | --- | --- | --- | --- |
| ***Maternal genotype*** | | |  |  |  |  |  |  |  |  |  |  |  |  |  |
| **rs1801133 MTHFR C677T** | | |  |  |  |  |  |  |  |  |  |  |  |  |  |
| CC | 1295 (47.7%) | 104 (49.3%) | 1 | 1309 (47.6%) | 89 (50.3%) | 1 | 1257 (47.5%) | 141 (51.3%) | 1 | 1335 (47.9%) | 64 (45.4%) | 1 | 1259 (47.5%) | 47 (51.1%) | 1 |
| CT | 1149 (42.3%) | 87 (41.2%) | 0.93  (0.69-1.25) | 1168 (42.5%) | 68 (38.4%) | 0.84  (0.60-1.17) | 1130 (42.7%) | 105 (38.2%) | 0.83  (0.63-1.08) | 1168 (41.9%) | 68 (48.2%) | 1.21 (0.85-1.73) | 1125 (42.5%) | 32 (34.8%) | 0.75 (0.47-1.20) |
| TT | 271 (10%) | 20 (9.5%) | 0.89  (0.54-1.47) | 271 (9.9%) | 20 (11.3%) | 1.05  (0.63-1.75) | 261 (9.9%) | 29 (10.5%) | 0.98  (0.64-1.50) | 282 (10.1%) | 9 (6.4%) | 0.65 (0.32-1.33) | 265 (10%) | 13 (14.1%) | 1.33 (0.70-2.52) |
| **rs1801131 MTHFR A1298C** | | |  |  |  |  |  |  |  |  |  |  |  |  |  |
| AA | 1323 (48.7%) | 88 (41.9%) | 1 | 1331 (48.4%) | 79 (44.9%) | 1 | 1267 (47.9%) | 142 (50.9%) | 1 | 1352 (48.5%) | 59 (41.5%) | 1 | 1276 (48.2%) | 43 (47.3%) | 1 |
| AC | 1169 (43%) | 96 (45.7%) | 1.21  (0.89-1.64) | 1183 (43%) | 82 (46.6%) | 1.15  (0.83-1.59) | 1160 (43.8%) | 104 (37.3%) | 0.79  (0.61-1.04) | 1197 (42.9%) | 68 (47.9%) | 1.28 (0.90-1.84) | 1146 (43.2%) | 40 (44%) | 1.01 (0.65-1.58) |
| CC | 227 (8.3%) | 26 (12.4%) | ***1.80***  ***(1.13-2.88)*** | 238 (8.6%) | 15 (8.5%) | 1.11  (0.62-1.97) | 220 (8.3%) | 33 (11.8%) | 1.28  (0.85-1.93) | 238 (8.5%) | 15 (10.6%) | 1.40 (0.78-2.51) | 228 (8.6%) | 8 (8.8%) | 1.10 (0.50-2.39) |
| **rs1805087 MTR A2756G** | | |  |  |  |  |  |  |  |  |  |  |  |  |  |
| AA | 1749 (65.2%) | 139 (66.2%) | 1 | 1776 (65.3%) | 111 (64.2%) | 1 | 1718 (65.8%) | 167 (59.4%) | 1 | 1797 (65.3%) | 91 (64.1%) | 1 | 1712 (65.4%) | 57 (63.3%) | 1 |
| AG | 844 (31.5%) | 63 (30%) | 0.95  (0.69-1.30) | 852 (31.3%) | 55 (31.8%) | 1.04  (0.74-1.46) | 804 (30.8%) | 103 (36.7%) | 1.32  (1.02-1.72) | 862 (31.3%) | 45 (31.7%) | 1.02 (0.71-1.48) | 818 (31.2%) | 29 (32.2%) | 1.08 (0.68-1.71) |
| GG | 90 (3.4%) | 8 (3.8%) | 1.22  (0.57-2.61) | 91 (3.3%) | 7 (4%) | 1.34  (0.60-3.03) | 87 (3.3%) | 11 (3.9%) | 1.32  (0.69-2.53) | 92 (3.3%) | 6 (4.2%) | 1.35 (0.57-3.17) | 88 (3.4%) | 4 (4.4%) | 1.46 (0.50-4.27) |
| **rs1801394 MTRR A66G** | | |  |  |  |  |  |  |  |  |  |  |  |  |  |
| CC | 612 (22.6%) | 45 (21.5%) | 1 | 610 (22.3%) | 46 (26.3%) | 1 | 591 (22.5%) | 64 (23.1%) | 1 | 629 (22.7%) | 28 (19.6%) | 1 | 580 (22%) | 23 (25.6%) | 1 |
| CT | 1325 (49%) | 103 (49.3%) | 1.01  (0.70-1.46) | 1356 (49.6%) | 72 (41.1%) | 0.67  (0.45-0.98) | 1291 (49.1%) | 136 (49.1%) | 0.96  (0.70-1.32) | 1357 (49%) | 71 (49.7%) | 1.14 (0.73-1.79) | 1302 (49.3%) | 42 (46.7%) | 0.78 (0.46-1.32) |
| TT | 766 (28.3%) | 61 (29.2%) | 1.04  (0.69-1.56) | 770 (28.1%) | 57 (32.6%) | 0.94  (0.62-1.42) | 750 (28.5%) | 77 (27.8%) | 0.92  (0.65-1.31) | 783 (28.3%) | 44 (30.8%) | 1.21 (0.74-1.97) | 757 (28.7%) | 25 (27.8%) | 0.80 (0.44-1.44) |
| **rs2236225 MTHFD1 G1958A** | | |  |  |  |  |  |  |  |  |  |  |  |  |  |
| CC | 876 (32.2%) | 71 (34%) | 1 | 888 (32.2%) | 59 (34.1%) | 1 | 864 (32.6%) | 81 (28.9%) | 1 | 896 (32.1%) | 51 (35.9%) | 1 | 862 (32.5%) | 30 (32.6%) | 1 |
| CT | 1353 (49.7%) | 108 (51.7%) | 0.94  (0.68-1.29) | 1377 (50%) | 83 (48%) | 0.86  (0.60-1.21) | 1316 (49.7%) | 144 (51.4%) | 1.15  (0.86-1.53) | 1397 (50.1%) | 64 (45.1%) | 0.78 (0.54-1.14) | 1315 (49.6%) | 48 (52.2%) | 0.96 (0.60-1.55) |
| TT | 492 (18.1%) | 30 (14.4%) | 0.74  (0.47-1.16) | 491 (17.8%) | 31 (17.9%) | 0.96  (0.61-1.51) | 467 (17.6%) | 55 (19.6%) | 1.22  (0.85-1.75) | 495 (17.8%) | 27 (19%) | 0.93 (0.57-1.50) | 473 (17.8%) | 14 (15.2%) | 0.84 (0.44-1.62) |
| **rs1801198 TCN2 C766G** | | |  |  |  |  |  |  |  |  |  |  |  |  |  |
| CC | 783 (29.3%) | 58 (27.9%) | 1 | 791 (29.2%) | 50 (28.7%) | 1 | 765 (29.4%) | 76 (27.3%) | 1 | 789 (28.8%) | 52 (36.6%) | 1 | 762 (29.3%) | 24 (26.4%) | 1 |
| CG | 1337 (50%) | 104 (50%) | 1.10  (0.78-1.54) | 1346 (49.7%) | 95 (54.6%) | 1.18  (0.82-1.69) | 1294 (49.8%) | 145 (52.2%) | 1.11  (0.83-1.49) | 1374 (50.1%) | 67 (47.2%) | 0.73 (0.50-1.06) | 1300 (49.9%) | 44 (48.4%) | 1.12 (0.67-1.88) |
| GG | 554 (20.7%) | 46 (22.1%) | 1.20  (0.79-1.80) | 570 (21.1%) | 29 (16.7%) | 0.87  (0.54-1.40) | 542 (20.8%) | 57 (20.5%) | 1.07  (0.74-1.54) | 577 (21.1%) | 23 (16.2%) | ***0.60 (0.36-0.99)*** | 543 (20.8%) | 23 (25.3%) | 1.58 (0.87-2.87) |
| ***Paternal Genotype*** | | |  |  |  |  |  |  |  |  |  |  |  |  |  |
| **rs1801133 MTHFR C677T** | | |  |  |  |  |  |  |  |  |  |  |  |  |  |
| CC | 939 (47.8%) | 72 (45.9%) | 1 | 964 (48.3%) | 47 (37%) | 1 | 915 (47.8%) | 95 (46.8%) | 1 | 958 (47.8%) | 53 (45.7%) | 1 | 900 (47.1%) | 36 (50.7%) | 1 |
| CT | 850 (43.3%) | 66 (42%) | 1.00  (0.70-1.42) | 851 (42.7%) | 65 (51.2%) | ***1.60***  ***(1.08-2.39)*** | 825 (43.1%) | 90 (44.3%) | 0.99  (0.73-1.35) | 864 (43.1%) | 52 (44.8%) | 1.04 (0.70-1.54) | 829 (43.4%) | 29 (40.8%) | 0.85 (0.51-1.42) |
| TT | 175 (8.9%) | 19 (12.1%) | 1.44  (0.84-2.48) | 179 (9%) | 15 (11.8%) | 1.80  (0.97-3.33) | 176 (9.2%) | 18 (8.9%) | 0.86  (0.50-1.47) | 183 (9.1%) | 11 (9.5%) | 0.99 (0.51-1.94) | 180 (9.4%) | 6 (8.5%) | 0.84 (0.34-2.05) |
| **rs1801131 MTHFR A1298C** | | |  |  |  |  |  |  |  |  |  |  |  |  |  |
| AA | 950 (48%) | 68 (42%) | 1 | 952 (47.2%) | 66 (52%) | 1 | 921 (47.5%) | 96 (47.8%) | 1 | 955 (47.2%) | 63 (53.4%) | 1 | 924 (47.8%) | 30 (43.5%) | 1 |
| AC | 845 (42.7%) | 80 (49.4%) | 1.34  (0.95-1.89) | 877 (43.5%) | 48 (37.8%) | 0.79  (0.53-1.16) | 836 (43.1%) | 88 (43.8%) | 1.03  (0.76-1.41) | 874 (43.2%) | 51 (43.2%) | 0.89 (0.61-1.31) | 835 (43.2%) | 32 (46.4%) | 1.20 (0.72-2.02) |
| CC | 185 (9.3%) | 14 (8.6%) | 1.08  (0.59-1.99) | 186 (9.2%) | 13 (10.2%) | 1.02  (0.55-1.92) | 182 (9.4%) | 17 (8.5%) | 0.94  (0.54-1.62) | 195 (9.6%) | 4 (3.4%) | ***0.32 (0.12-0.89)*** | 176 (9.1%) | 7 (10.1%) | 1.15 (0.48-2.75) |
| **rs1805087 MTR A2756G** | | |  |  |  |  |  |  |  |  |  |  |  |  |  |
| AA | 1186 (62.9%) | 98 (62%) | 1 | 1205 (62.7%) | 79 (64.2%) | 1 | 1150 (62.3%) | 132 (67.3%) | 1 | 1223 (63.2%) | 61 (55.5%) | 1 | 1155 (62.6%) | 44 (62%) | 1 |
| AG | 623 (33%) | 54 (34.2%) | 1.10  (0.77-1.56) | 638 (33.2%) | 39 (31.7%) | 0.98  (0.65-1.46) | 620 (33.6%) | 57 (29.1%) | 0.80  (0.57-1.11) | 631 (32.6%) | 46 (41.8%) | 1.48 (0.99-2.19) | 612 (33.2%) | 22 (31%) | 0.99 (0.58-1.70) |
| GG | 78 (4.1%) | 6 (3.8%) | 0.89  (0.37-2.13) | 79 (4.1%) | 5 (4.1%) | 0.91  (0.35-2.38) | 77 (4.2%) | 7 (3.6%) | 0.83  (0.37-1.86) | 81 (4.2%) | 3 (2.7%) | 0.77 (0.24-2.51) | 77 (4.2%) | 5 (7%) | 1.66 (0.61-4.51) |
| **rs1801394 MTRR A66G** | | |  |  |  |  |  |  |  |  |  |  |  |  |  |
| CC | 446 (22.4%) | 30 (18.6%) | 1 | 456 (22.6%) | 20 (15.7%) | 1 | 436 (22.5%) | 40 (19.2%) | 1 | 454 (22.4%) | 22 (18.3%) | 1 | 427 (22.1%) | 14 (19.7%) | 1 |
| CT | 950 (47.8%) | 83 (51.6%) | 1.34  (0.86-2.08) | 969 (47.9%) | 64 (50.4%) | 1.55  (0.92-2.62) | 916 (47.3%) | 116 (55.8%) | 1.34  (0.92-1.97) | 972 (47.9%) | 61 (50.8%) | 1.27 (0.77-2.10) | 936 (48.4%) | 38 (53.5%) | 1.24 (0.66-2.36) |
| TT | 591 (29.7%) | 48 (29.8%) | 1.16  (0.71-1.87) | 596 (29.5%) | 43 (33.9%) | 1.56  (0.89-2.72) | 586 (30.2%) | 52 (25%) | 0.93  (0.60-1.43) | 602 (29.7%) | 37 (30.8%) | 1.24 (0.72-2.13) | 572 (29.6%) | 19 (26.8%) | 0.87 (0.42-1.79) |
| **rs2236225 MTHFD1 G1958A** | | |  |  |  |  |  |  |  |  |  |  |  |  |  |
| CC | 644 (32.3%) | 53 (32.3%) | 1 | 657 (32.4%) | 40 (31.2%) | 1 | 626 (32.1%) | 70 (34.5%) | 1 | 658 (32.3%) | 39 (32.8%) | 1 | 628 (32.2%) | 23 (31.9%) | 1 |
| CT | 956 (48%) | 77 (47%) | 0.95  (0.66-1.38) | 970 (47.8%) | 63 (49.2%) | 1.03  (0.68-1.56) | 941 (48.2%) | 91 (44.8%) | 0.88  (0.63-1.23) | 966 (47.4%) | 67 (56.3%) | 1.18 (0.78-1.77) | 929 (47.7%) | 41 (56.9%) | 1.17 (0.68-1.99) |
| TT | 392 (19.7%) | 34 (20.7%) | 1.05  (0.66-1.65) | 401 (19.8%) | 25 (19.5%) | 1.01  (0.60-1.70) | 384 (19.7%) | 42 (20.7%) | 0.96  (0.64-1.44) | 413 (20.3%) | 13 (10.9%) | ***0.52 (0.28-0.99)*** | 392 (20.1%) | 8 (11.1%) | 0.52 (0.23-1.19) |
| **rs1801198 TCN2 C766G** | | |  |  |  |  |  |  |  |  |  |  |  |  |  |
| CC | 592 (30.8%) | 51 (31.9%) | 1 | 613 (31.3%) | 30 (24.4%) | 1 | 582 (30.9%) | 60 (30.5%) | 1 | 607 (30.8%) | 36 (32.1%) | 1 | 587 (31.3%) | 16 (22.9%) | 1 |
| CG | 961 (50.1%) | 79 (49.4%) | 0.95  (0.65-1.38) | 973 (49.7%) | 67 (54.5%) | 1.40  (0.89-2.19) | 942 (50.1%) | 98 (49.7%) | 0.97  (0.69-1.37) | 981 (49.8%) | 59 (52.7%) | 0.99 (0.64-1.52) | 926 (49.4%) | 39 (55.7%) | 1.48 (0.81-2.71) |
| GG | 367 (19.1%) | 30 (18.8%) | 0.92  (0.57-1.49) | 371 (19%) | 26 (21.1%) | 1.42  (0.82-2.45) | 357 (19%) | 39 (19.8%) | 1.10  (0.72-1.69) | 380 (19.3%) | 17 (15.2%) | 0.77 (0.42-1.38) | 363 (19.3%) | 15 (21.4%) | 1.48 (0.71-3.08) |

**Supplementary Table3: Maternal dietary intake preconception and at 15±1 weeks’ gestation, and blood biochemistry in the Adelaide 325 cohort**

| **Dietary intake** | **N** | **Folate**  **(nmol/L)** | **P** | **B12**  **(pmol/L)** | **P** | **Hcy**  **(μmol/L)** | **P** |
| --- | --- | --- | --- | --- | --- | --- | --- |
| **Fast food – PC**  **(serves/week)** | | |  |  |  |  |  |
| 0 to ≤ 2 | 17 | 31.6 (26.3, 37.8) | ref | 258.4 (212.8, 313.9) | ref | 5.86 (5.26, 6.54) | ref |
| > 2 to ≤ 4 | 112 | 29.2 (26.4, 32.4) | 0.379 | 257.6 (230.7, 287.7) | 0.974 | 6.00 (5.64, 6.38) | 0.668 |
| > 4 | 121 | 29.6 (26.7, 32.8) | 0.461 | 246.8 (220.7, 275.9) | 0.626 | 6.09 (5.72, 6.49) | 0.479 |
| **Green vegetables - PC**  **(serves/day)** | | |  |  |  |  |  |
| < 1 | 245 | 28.9 (26.6, 31.4) | ref | 247.7 (228.5, 268.5) | ref | 6.06 (5.78, 6.35) | ref |
| ≥ 1 | 81 | 27.3 (24.5, 30.4) | 0.222 | 270.3 (243.6, 299.9) | 0.052 | 6.11 (5.76, 6.49) | 0.731 |
| **Fruit – PC**  **(serves/day)** | | |  |  |  |  |  |
| < 1 | 226 | 28.6 (26.3, 31.0) | ref | 250.6 (231.2, 271.5) | ref | 6.08 (5.81, 6.37) | ref |
| ≥ 1 | 100 | 28.3 (25.4, 31.6) | 0.824 | 261.4 (234.9, 290.8) | 0.351 | 6.01 (5.65, 6.39) | 0.633 |
| **Fast food - 15 weeks’**  **(serves/week)** | | |  |  |  |  |  |
| 0 to ≤ 2 | 29 | 28.0 (24.1, 32.4) | ref | 245.7 (209.0, 288.8) | ref | 6.22 (5.68, 6.81) | ref |
| > 2 to ≤ 4 | 133 | 28.9 (26.1, 31.9) | 0.640 | 254.7 (228.7, 283.7) | 0.623 | 6.06 (5.70, 6.43) | 0.517 |
| > 4 | 89 | 30.9 (27.7, 34.3) | 0.165 | 251.5 (224.1, 282.2) | 0.762 | 5.96 (5.58, 6.36) | 0.317 |
| **Green vegetables - 15 weeks’**  **(serves/day)** | | |  |  |  |  |  |
| < 1 | 232 | 28.8 (26.5, 31.4) | ref | 245.6 (226.1, 266.7) | ref | 6.09 (5.81, 6.39) | ref |
| ≥ 1 | 94 | 28.0 (25.3, 30.9) | 0.512 | 268.5 (243.7, 295.9) | ***0.037*** | 6.02 (5.69, 6.37) | 0.608 |
| **Fruit - 15 weeks’**  **(serves/day)** | | |  |  |  |  |  |
| < 1 | 143 | 28.0 (25.5, 30.7) | ref | 252.3 (230.6, 276.2) | ref | 6.15 (5.84, 6.48) | ref |
| ≥ 1 | 183 | 29.0 (26.5, 31.6) | 0.385 | 252.7 (231.9, 275.4) | 0.970 | 6.01 (5.72, 6.31) | 0.285 |

*Data are adjusted for maternal age, BMI, smoking and folic acid supplementation at 15 weeks’. PC: preconception. Data are presented as adjusted marginal means (95% CI); P values in bold are statistically significant.*

**Supplementary Table 2: Maternal dietary intake preconception and at 15±1 weeks’ gestation, and birth outcomes in the Adelaide and Auckland 3196 cohort**

| **Dietary Intake** | **N** | **Birthweight (g)^#^** | **P** | **Gestational age (weeks)*** | **P** |
| --- | --- | --- | --- | --- | --- |
| **Fast food – PC (serves/week)** | | |  |  |  |
| none to ≤2x | 479 | 3270 (3202, 3338) | ref | 39.2 (38.7, 39.7) | ref |
| >2 to ≤ 4 | 945 | 3323 (3267, 3380) | 0.078 | 39.1 (38.7, 39.4) | 0.496 |
| >4x | 561 | 3272 (3210, 3335) | 0.944 | 39.1 (38.7, 39.5) | 0.648 |
| **Green vegetables – PC (serves/day)** | | |  |  |  |
| <1x | 1625 | 3290 (3249, 3331) | ref | 39.1 (38.8, 39.4) | ref |
| ≥1x | 1571 | 3312 (3269, 3356) | 0.232 | 39.0 (38.7, 39.3) | 0.497 |
| **Fruit – PC (serves/day)** | |  |  |  |  |
| <1x | 1251 | 3298 (3255, 3340) | ref | 39.0 (38.7, 39.3) | ref |
| ≥1x | 1945 | 3301 (3259, 3345) | 0.846 | 39.1 (38.8, 39.4) | 0.728 |
| **Fast food - 15 weeks’ (serves/week)** | | |  |  |  |
| none to ≤2 | 548 | 3297 (3234, 3362) | ref | 38.9 (38.5, 39.4) | ref |
| >2 to ≤4 | 1013 | 3317 (3263, 3372) | 0.479 | 39.1 (38.8, 39.5) | 0.326 |
| >4 | 428 | 3265 (3201, 3330) | 0.385 | 39.2 (38.8, 39.7) | 0.257 |
| **Green vegetables - 15 weeks’ (serves/day)** | | |  |  |  |
| <1 | 1743 | 3295 (3254, 3336) | ref | 39.1 (38.8, 39.4) | ref |
| ≥1 | 1453 | 3305 (3263, 3349) | 0.567 | 38.9 (38.7, 39.2) | 0.173 |
| **Fruit - 15 weeks’ (serves/day)** | | |  |  |  |
| <1 | 817 | 3284 (3237, 3332) | ref | 39.0 (38.7, 39.4) | ref |
| ≥1 | 2379 | 3307 (3266, 3347) | 0.316 | 39.0 (38.8, 39.3) | 0.979 |

*PC: preconception. ^#^ Data are adjusted for maternal age, BMI, smoking, folate supplement, gestational age, and study center. * Data are adjusted for maternal age, BMI, smoking, folate supplement and study center. Data are presented as estimated marginal means (95% CI); P values in bold are statistically significant.*
